# Can the virtual reality intervention VR FestLab improve Danish studentś alcohol competences?

**DOI:** 10.1093/eurpub/ckac131.314

**Published:** 2022-10-25

**Authors:** J Guldager, SL Kjær, U Grittner, C Stock

**Affiliations:** 1 Department of Public Health, University of Southern Denmark, Esbjerg, Denmark; 2 Department of Physiotherapy, University College South Denmark, Esbjerg, Denmark; 3 Institute of Biometry and Clinical Epidemiology, Charité - Universitätsmedizin Berlin, Berlin, Germany; 4 Institute of Health and Nursing Science, Charité - Universitätsmedizin Berlin, Berlin, Germany

## Abstract

**Background:**

VR FestLab is a newly developed simulated “party” where the player is being offered alcoholic beverages while steering their own party experience in virtual reality. We evaluated the efficacy of VR FestLab on drinking refusal self-efficacy of Danish male and female 15-18-year-old students and tested user satisfaction. Secondary outcomes were drug refusal skills, knowledge/awareness, communication skills, social support willingness, susceptibility to peer pressures, and outcome expectations.

**Methods:**

The intervention consisted of 15 minutes of gameplay and 30 minutes of group/in class discussion. The intervention group played VR FestLab where the control group played the VR game “Oculus Quest - First Steps” (no educational content). In a cluster-randomised controlled trial schools were randomly assigned to the intervention/control group. 13 Danish schools were recruited containing 181 students in intervention/191 in control groups. Students completed a questionnaire before and immediately after the intervention. Data was analysed using mixed linear regression models.

**Results:**

50% of students found VR FestLab to be realistic, 57% would like to explore it further, and 43% would recommend it to friends. We found a small, but non-significant effect on drinking refusal self-efficacy favoring the intervention. No differences between the intervention and control group were observed for the secondary outcomes.

**Conclusions:**

VR FestLab should be used with higher frequency than in this trial and in combination with other evidence-based alcohol prevention interventions adolescents. Further research is needed to improve the effectiveness of VR FestLab.

**Key messages:**

• VR FestLab could be a valuable contribution to school-based alcohol prevention education but cannot stand alone.

• Future research should focus on the dose-effect relationship of the novel tool.

